# Study on the Passivation of Defect States in Wide-Bandgap Perovskite Solar Cells by the Dual Addition of KSCN and KCl

**DOI:** 10.3390/nano15201602

**Published:** 2025-10-21

**Authors:** Min Li, Zhaodong Peng, Xin Yao, Jie Huang, Dawei Zhang

**Affiliations:** 1 College of Optical Electrical and Computer Engineering, University of Shanghai for Science and Technology, Shanghai 200093, China; 2 Zhejiang Institute of Medical Device Supervision and Testing, Hangzhou 310018, China; 3 College of Optical and Electronic Technology, China Jiliang University, Hangzhou 310018, China

**Keywords:** wide-bandgap perovskite, perovskite solar cells, defect passivation, interface engineering

## Abstract

Wide-bandgap (WBG) perovskite solar cells (PSCs) are critical for high-efficiency tandem photovoltaic devices, but their practical application is severely limited by phase separation and poor film quality. To address these challenges, this study proposes a dual-additive passivation strategy using potassium thiocyanate (KSCN) and potassium chloride (KCl) to synergistically optimize the crystallinity and defect state of WBG perovskite films. The selection of KSCN/KCl is based on their complementary functionalities: K^+^ ions occupy lattice vacancies to suppress ion migration, Cl^−^ ions promote oriented crystal growth, and SCN^−^ ions passivate surface defects via Lewis acid-base interactions. A series of KSCN/KCl concentrations (relative to Pb) were tested, and the effects of dual additives on film properties and device performance were systematically characterized using scanning electron microscopy (SEM), X-ray diffraction (XRD), X-ray photoelectron spectroscopy (XPS), photoluminescence (PL), space-charge-limited current (SCLC), current-voltage (*J-V*), and external quantum efficiency (EQE) measurements. Results show that the dual additives significantly enhance film crystallinity (average grain size increased by 27.0% vs. control), reduce surface roughness (from 86.50 nm to 24.06 nm), and passivate defects-suppressing non-radiative recombination and increasing electrical conductivity. For WBG PSCs, the champion device with KSCN (0.5 mol%) + KCl (1 mol%) exhibits a power conversion efficiency (*PCE*) of 16.85%, representing a 19.4% improvement over the control (14.11%), along with enhanced open-circuit voltage (*Voc*: +2.8%), short-circuit current density (*Jsc*: +6.7%), and fill factor (*FF*: +8.9%). Maximum power point (MPP) tracking confirms superior operational stability under illumination. This dual-inorganic-additive strategy provides a generalizable approach for the rational design of stable, high-efficiency WBG perovskite films.

## 1. Introduction

Perovskite/silicon tandem solar cells offer a promising pathway to maximize utilization of the solar spectrum, surpassing the theoretical efficiency limit of costs. Consequently, they have attracted significant research interest within the photovoltaic community. While perovskite solar cells demonstrate remarkable efficiency and processing advantages, researchers have explored various alternative photovoltaic technologies, each with distinct characteristics. Chalcogenide thin films (e.g., CIGS and CdTe) benefit from mature fabrication processes and proven long-term stability, yet face challenges in further efficiency improvement and cost reduction. Environmentally friendly perovskite-inspired materials (PIMs) offer promising lead-free alternatives but currently lag behind in efficiency performance. Engineered III-V nanostructures exhibit exceptional optoelectronic properties and precise bandgap tunability through quantum confinement effects, making them ideal for specialized applications [1], though their high fabrication costs and complex synthesis routes limit widespread adoption. In contrast, perovskite materials uniquely combine the advantages of these technologies: they possess bandgap tunability comparable to III-V semiconductors, solution processability similar to chalcogenide films, and efficiency potential surpassing PIMs, while maintaining relatively low fabrication costs. Nevertheless, perovskites remain exceptional candidates for tandem applications due to their superior bandgap tunability, high defect tolerance, and compatibility with low-cost fabrication. Thus, this work focuses on addressing critical challenges in wide-bandgap perovskite materials to advance their practical use in high-efficiency tandem solar cells. In recent years, considerable efforts have been devoted to enhancing the performance of perovskite/silicon tandem devices. Key strategies include implementing light-trapping structures via silicon substrate texturing [2,3,4], optimizing transparent conductive electrodes [5,6,7], incorporating functional interlayers [8,9,10], refining tunnel junction design [11,12,13], and improving perovskite film growth techniques [14,15]. Despite these advances, the further development of tandem architectures remains constrained by several intrinsic challenges associated with perovskite materials. However, the inherent stability limitations of perovskite materials and significant open-circuit voltage losses relative to theoretical limits severely constrain the further development of tandem solar cells.

The open-circuit voltage (V_OC_) of a tandem solar cell theoretically approaches the sum of the top and bottom cell V_OC_s, while the short-circuit current depends on the smaller of the two sub-cells. Consequently, the optimal bandgap width for a perovskite top cell stacked with silicon is 1.7–1.8 eV [16,17]. Currently, single-junction perovskite cells with ~1.5 eV bandgap have achieved V_OC_ losses as low as 0.3 V (E_g_/q-V_OC_) [18], while V_OC_ losses for ~1.7 eV bandgap perovskite solar cells mostly remain above 0.5 V. The primary causes of open-circuit voltage loss are twofold: (1) Wide-bandgap perovskites are prone to phase separation under operating conditions (exposure to light and heat), leading to higher V_OC_ loss and poorer stability [19,20]. (2) Higher defect concentrations arise at the interfaces between the wide-bandgap perovskite layer and the charge transport layers. Particularly, deep-level defects trap electrons or holes. These trapped carriers, unable to escape due to thermal activation, undergo non-radiative recombination with oppositely charged carriers. This process releases part of the electrical energy as phonons into the adjacent lattice, reducing the steady-state carrier density, diffusion length, and lifetime in the solar cell, thereby degrading performance. Moreover, these defects serve as primary reaction sites for water and oxygen, leading to device degradation and compromising the performance and stability of wide-bandgap perovskite solar cells [21,22]. Additionally, elevated defect state density exacerbates phase separation during device operation [23], further impairing device performance. Therefore, suppressing the formation of deep-level defect states in both the bulk and interfaces of wide-bandgap perovskite materials is an urgent task for fabricating highly efficient and stable perovskite/silicon tandem solar cells. Extensive research has confirmed that the morphological characteristics of WBG perovskite films, such as grain size, crystallinity, and defect state, are key factors in suppressing phase separation and directly determine the final device performance [24,25,26].

To address the above challenges, additive engineering has been established as a versatile and effective strategy to optimize WBG perovskite film quality. By introducing functional additives into precursor solutions, researchers can precisely regulate the nucleation and growth kinetics of perovskite crystals, thereby increasing grain size, reducing grain boundary density, and passivating surface/interface defects [27,28]. For instance, Zhao et al. [29] simultaneously introduced lead chloride (PbCl_2_) and phenethylammonium chloride (PEACl) into perovskites, which inhibited ion migration and promoted the formation of 2D perovskites, drastically reducing the open-circuit voltage deficit in WBG PSCs and achieving a PCE exceeding 20%. Jeong et al. [30] introduced formate anions (HCOO^−^) into perovskites, producing films with high crystallinity and large grain size, resulting in a certified PCE of 25.2%. Kong et al. [31] used potassium (4-chlorophenyl) trifluoroborate (4-ClPTFBK) as an additive, which improved film crystallinity, reduced defect density, and effectively suppressed non-radiative recombination. Despite these advances, single-additive strategies often suffer from trade-offs; for example, Cl^−^-based additives effectively enhance crystallinity but show limited ability to suppress long-term ion migration, while SCN^−^-based additives improve grain size but may introduce residual organic impurities that degrade stability. In a recent study, Meng et al. [32] presented an effective approach combining molecular additives with interfacial passivation layers for performance enhancement in WBG perovskites. Complementary to such multi-component strategies, our work explores a distinct pathway that relies exclusively on the coordinated use of dual inorganic additives, KSCN and KCl, to simultaneously modulate crystallization kinetics and passivate bulk defects. Compared to existing approaches, our KSCN + KCl dual-additive strategy demonstrates distinct advantages: it avoids the environmental concerns of lead-containing additives, simplifies the fabrication process compared to complex organic molecules or multi-step passivation schemes, and maintains cost-effectiveness through the use of commercially available inorganic salts. While recent hybrid strategies combining additives with external passivation layers have shown impressive results, our work proves that comparable performance (PCE > 16.8% with excellent stability) can be achieved through a streamlined, additive-only approach, offering a more industrially viable pathway for wide-bandgap perovskite development. Additionally, few studies have systematically explored the synergistic effects of dual inorganic additives (e.g., alkali metal halides) on WBG perovskite film formation and device performance, leaving a critical gap in the development of high-efficiency and stable WBG PSCs.

Herein, we propose a dual-additive passivation strategy utilizing potassium thiocyanate (KSCN) and potassium chloride (KCl) to concurrently optimize the crystallinity and defect state of wide-bandgap (WBG) perovskite films, with the selection of these two inorganic additives driven by their distinct yet complementary functionalities: firstly, potassium ions (K^+^), featuring a small ionic radius, can occupy vacant lattice sites within the perovskite structure to suppress ion migration, and secondly, chloride ions (Cl^−^) facilitate oriented crystal growth while thiocyanate ions (SCN^−^) passivate surface defects via Lewis acid-base interactions: their synergistic combination is thus expected to achieve the integrated effect of “crystallinity enhancement coupled with defect passivation”. To systematically validate this strategy, we examine how varying KSCN/KCl concentration ratios influence the key properties of WBG perovskite films, including crystalline structure (characterized via X-ray diffraction, XRD), surface morphology (observed using scanning electron microscopy, SEM), and defect chemistry (analyzed by X-ray photoelectron spectroscopy, XPS, and photoluminescence spectroscopy, PL); for the corresponding WBG perovskite solar cells (PSCs), their photovoltaic performance is assessed through current-voltage (*J-V*) measurements. Central to this work is the objective to elucidate the synergistic interaction mechanisms governing the interplay between KSCN/KCl additives and WBG perovskite lattices, alongside the pursuit of fabricating WBG PSCs with a power conversion efficiency (*PCE*) exceeding 16.8%. This study provides a generalizable dual-inorganic-additive strategy for the rational design of stable WBG perovskite films.

## 2. Experimental Section

### 2.1. Materials and Chemical Reagents

Cesium iodide (CsI, 99%), lead bromide (PbBr_2_, 99.9%), formamidinium iodide (FAI, 99.999%), lead iodide (PbI_2_, 99.999%), fullerene (C_60_, 99%), bathocuproine (BCP, 98%), potassium thiocyanate (KSCN, 98.5%), potassium chloride (KCl, 99.77%), ethyl acetate (EA, 99.8%), silver (Ag, 99.999%), anhydrous ethanol (AR), isopropyl alcohol (IPA, 99.8%), N,N-dimethylformamide (DMF, 99.8%), dimethyl sulfoxide (DMSO, 99.9%), ITO conductive glass (7–9 Ω/sq), ITO cleaner (DECON90).

All the ingredients mentioned were procured as follows: Cesium iodide (CsI), lead bromide (PbBr_2_) and fullerene (C_60_) were provided by Xi’an Baolite; formamidinium iodide (FAI), lead iodide (PbI_2_), dimethyl sulfoxide (DMSO) and ITO conductive glass were purchased from Liaoning Youxuan; bathocuproine (BCP), ethyl acetate (EA), silver (Ag), N,N-dimethylformamide (DMF) and isopropyl alcohol (IPA) were supplied by Shanghai Aladdin; potassium thiocyanate (KSCN) was obtained from Shanghai General Reagent; potassium chloride (KCl) was procured from Bide Pharmatech; anhydrous ethanol was provided by Sinopharm Group; ITO cleaner (DECON90) was purchased from Decon Laboratories (East Sussex, UK).

### 2.2. Preparation of Precursor Solution with Additives

The precursor solution with additives was prepared as follows: CsI (67.54 mg), FAI (179.8 mg), PbI_2_ (329.6 mg), and PbBr_2_ (214.68 mg) were dissolved in a mixed solvent of DMF (800 µL) and DMSO (200 µL) under vigorous stirring to form 1 mL of perovskite precursor solution with a concentration of 1.3 mol/L. Separate solutions of KSCN and KCl at concentrations of 10 mg/mL and 20 mg/mL were prepared using a 4:1 (v:v) DMF: DMSO solvent mixture. These additive solutions were then incorporated into the perovskite precursor solution to achieve the following molar ratios (relative to Pb): KSCN (0.3 mol%) + KCl (0.5 mol%), KSCN (0.3 mol%) + KCl (1 mol%), KSCN (0.5 mol%) + KCl (0.5 mol%), and KSCN (0.5 mol%) + KCl (1 mol%), resulting in the target additive concentration perovskite precursor solutions.

### 2.3. Fabrication of Perovskite Solar Cells

**Figure 1:**
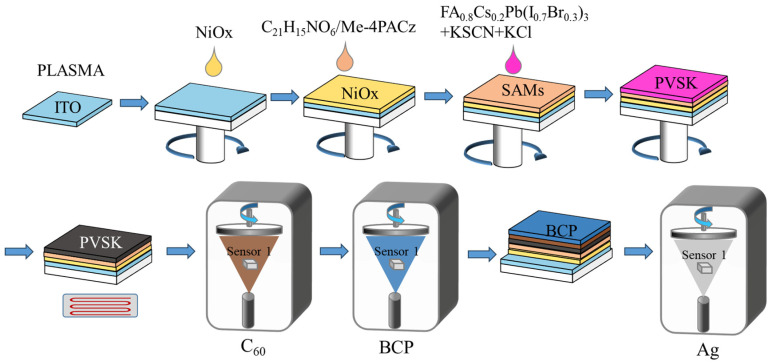
The fabrication flowchart of the perovskite solar cell.

The fabrication flowchart of the perovskite solar cell is shown in Figure 1.

1. ITO Substrate Cleaning: This experiment utilized square ITO substrates with a side length of 2.5 cm. The substrates were placed on a PTFE cleaning rack and subjected to ultrasonic cleaning for 15 min using DECON-90, deionized water, anhydrous ethanol, and isopropyl alcohol. They were then dried using a nitrogen gun. Subsequently, the ITO surface was treated with PLASMA for 15 min to enhance its wettability.

2. Hole Transport Layer Preparation: The NiOx layer was prepared via spin coating. NiOx reagent was dissolved in deionized water to form a 15 mg/mL NiO_x_ solution. 70 µL of the NiOx aqueous solution was dispensed onto the ITO surface. Spin coating was performed at 1500 rpm for 30 s, followed by annealing on a constant-temperature heating platform at 250 °C for 30 min.

Mixed SAMs Layer: Dissolve triphenylamine tricarboxylate and Me-4PACz separately in ethanol solutions at 1 mg/mL concentrations, then mix uniformly in a 1:3 ratio. Dispense 70 µL of the mixed solution onto the NiO_x_ layer and spin-coat at 3000 rpm for 30 s. Subsequently, anneal at 100 °C on a constant-temperature heating platform for 10 min.

3. Preparation of perovskite light-absorbing layer: The WBG perovskite precursor solution concentration was 1.3 mol/L. For spin-coating, 100 µL of precursor solution with different additive concentrations was used. The spin-coating program consisted of a first step at 1000 rpm for 5 s with an acceleration of 500 rpm/s to ensure uniform distribution of the precursor solution on the ITO substrate, followed by a second step at 3000 rpm for 30 s with an acceleration of 1500 rpm/s. Anti-solvent engineering was applied by dripping 250 µL of ethyl acetate (EA) 15 s before the end of the second step, resulting in a wet perovskite film. Subsequently, the film was annealed on a hotplate at 100 °C for 20 min to form the perovskite absorber layer with the respective KSCN and KCl concentrations (relative to Pb).

4. C_60_/BCP Electronic Transport Layer Preparation: Under a vacuum of 1 × 10^−5^ Pa, sequentially deposit 50 nm of C_60_ and 8 nm of BCP at a rate of 0.3 Å/s.

5. Metal Electrode Layer Preparation: Under a vacuum of 1 × 10^−5^ Pa, deposit 140 nm of Ag at a rate of 1 Å/s.

**Figure 2:**
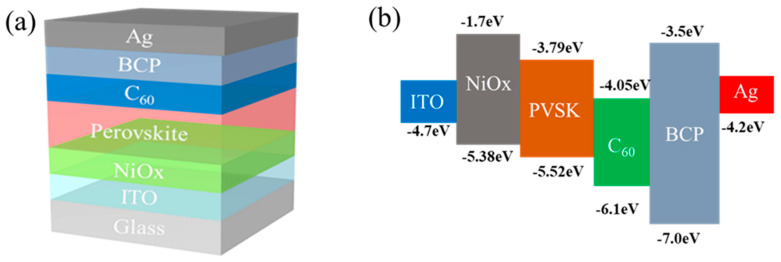
(**a**) The structure of the perovskite solar cell; (**b**) The energy level alignment of each layer.

The structure of the perovskite solar cell and the energy level alignment of each layer are illustrated in Figure 2 below.

### 2.4. Instruments and Measurements

The fabrication of perovskite films and devices was performed with the following equipment: ITO conductive glass was cleaned using an ultrasonic cleaner (Shenzhen Jiemeng Cleaning, JP-040S, Shenzhen, China) and a plasma cleaner (Jiangyin Xirui Electronics, PDC-002, Jiangyin, China); perovskite precursor solutions were prepared using an analytical balance (Shanghai Tianmei, FA1204B, Shanghai, China) and a constant-temperature magnetic stirrer (Shanghai Meiyingpu, 08-2G, Shanghai, China); perovskite films were deposited via a spin coater (Jiangsu Raybo Scientific Instruments Co., Ltd., EZ4, Jiangyin, China) and annealed on a constant-temperature heating stage (Shenzhen Xincheng, JR-1515, Shenzhen, China); the Ag electrode was deposited using a vacuum evaporation system (Beijing Taike Nuo, ZHDS400, Beijing, China); all air-sensitive operations were conducted in a glove box (Shanghai Mikrouna, Univeresal, Shanghai, China).

The morphology of WBG perovskite films was observed using a scanning electron microscope (ZEISS, sigma 300, Jena, Germany, EHT = 3.00 kV, WD = 5.5 mm) with operating parameters carefully selected to minimize electron-beam-induced damage to the perovskite samples while maintaining sufficient image resolution and a confocal microscope (ZEISS, Smartproof 5). The crystalline structure of the films was characterized by an X-ray diffractometer (PANalytical, Xpert Powder). The elemental composition and chemical state of the films were analyzed via an X-ray photoelectron spectrometer (THERMO SCIENTIFIC, K-ALPHA, Waltham, MA, USA). For both XRD and XPS measurements, the operating conditions were optimized to prevent radiation damage to the delicate perovskite structure while ensuring adequate signal quality. The steady-state and transient photoluminescence properties of the films were tested using a steady-state/transient fluorescence spectrometer (EDINBURGH INSTRUMENTS, FLS1000, Livingston, UK). The optical transmittance of the films was measured with an ultraviolet-visible spectrophotometer (PerkinElmer, Lambda365, Waltham, MA, USA).

The photovoltaic performance of WBG PSCs was evaluated using a current-voltage (*J-V*) test system (Beijing Saifan Optoelectronics, SCS020, Beijing, China) and an external quantum efficiency (EQE) test system (Beijing Saifan Optoelectronics, 7-SCSpec).

## 3. Results and Discussion

### 3.1. Effect of KSCN and KCl Dual Additives on the Crystallization Properties of WBG Perovskite Films

**Figure 3:**
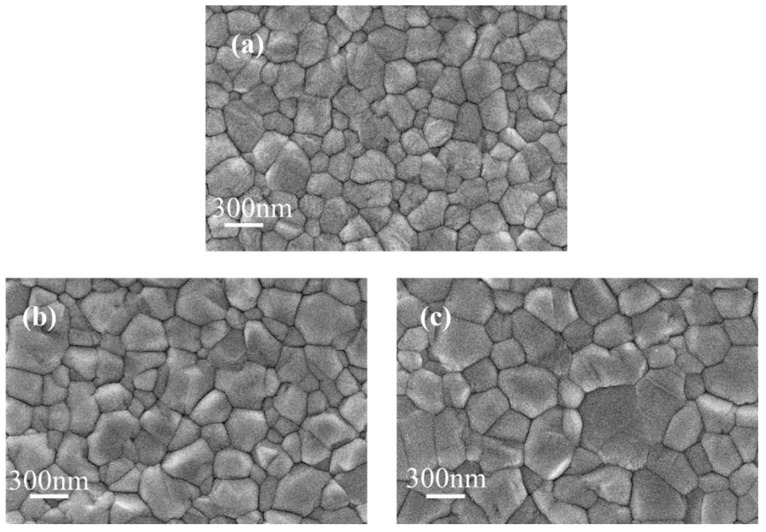
Surface SEM images before and after the introduction of additives; (**a**) No additives introduced; (**b**) KSCN (0.3 mol%) + KCl (0.5 mol%) introduced; (**c**) KSCN (0.5 mol%) + KCl (1 mol%) introduced.

**Figure 4:**
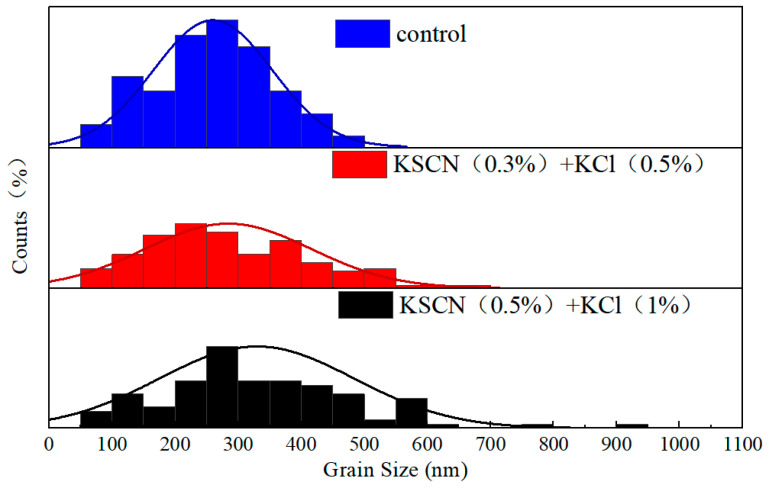
Distribution of grain size under different conditions.

The influence of KSCN and KCl dual additives on the crystallinity of WBG perovskite films was first examined by scanning electron microscopy (SEM). Grain size analysis was performed using Nano Measurer software (version: 1.2), which operates by identifying grain boundaries through grayscale threshold segmentation and calculating the maximum Feret diameter—defined as the longest distance between any two parallel planes bounding the grain profile. This parameter provides a representative dimensional characterization of irregularly shaped grains. Multiple regions were selected for measurement to ensure statistical significance. The results are shown in Figure 3. The SEM images in Figure 3 reveal that the incorporation of KSCN + KCl additives not only promotes grain growth while maintaining a similar morphological structure. Statistical analysis of the grain size distribution under different conditions, performed using Nano Measurer software (Figure 4), reveals a significant increase in average grain size from 259.9 nm for the control film to 329.6 nm for the film with KSCN (0.5 mol%) + KCl (1 mol%) additives, representing a 27.0% enhancement. The observed improvement in optoelectronic performance can be mainly attributed to this grain size enlargement and the consequent reduction in grain boundary density, rather than substantial changes in film morphology. The grain size in the control film (without additives) ranged from 50 nm to 500 nm, with an average size of 259.9 nm. For the film with KSCN (0.3 mol%) + KCl (0.5 mol%), the grain size range expanded to 50 nm~700 nm, and the average size increased to 284.2 nm, representing a 9.7% enhancement. The film with KSCN (0.5 mol%) + KCl (1 mol%) exhibited a further increase, ranging from 500 nm to 1000 nm with an average of 329.6 nm, corresponding to a 27.0% increase relative to the control. These results demonstrate that the KSCN + KCl dual additives effectively promote grain growth, reducing defect density at grain boundaries and consequently improving film quality. The promoting effect of the dual additives on grain size growth is diminished. After K^+^ ions occupy the lattice vacancies, atomic diffusion at the grain boundaries is slowed down, thereby restricting grain growth.

**Figure 5:**
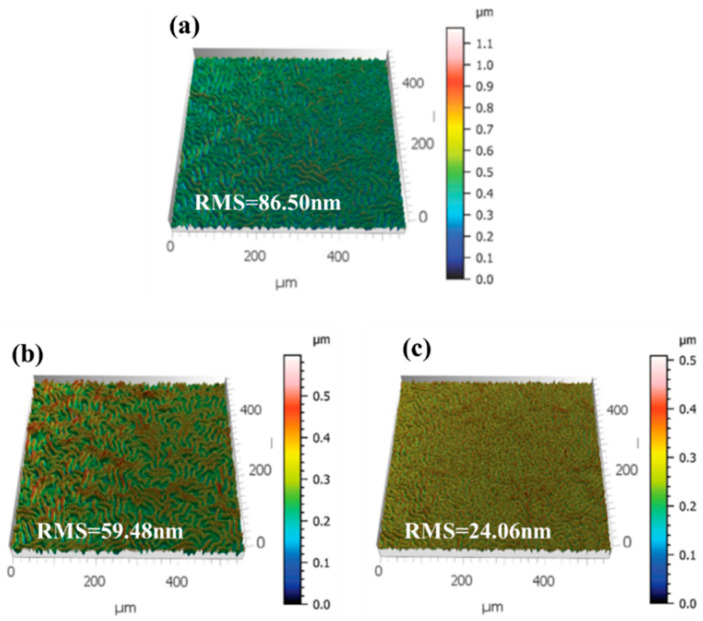
Surface roughness before and after introduction of additives. (**a**) no additive introduced; (**b**) introduction of KSCN (0.3 mol%) + KCl (0.5 mol%); (**c**) KSCN (0.5 mol%) + KCl (1 mol%).

The surface roughness of the WBG perovskite films, another indicator of film quality, was also investigated (Figure 5). Measurements indicated a significant reduction in surface roughness from 86.50 nm for the untreated film to 24.06 nm for the film with dual additives. The introduction of the dual additives further enhances the smoothness of the perovskite film. This suggests that K^+^ and Cl^−^ ions contribute to vacancy filling, further reducing defect density and suppressing phase separation.

**Figure 6:**
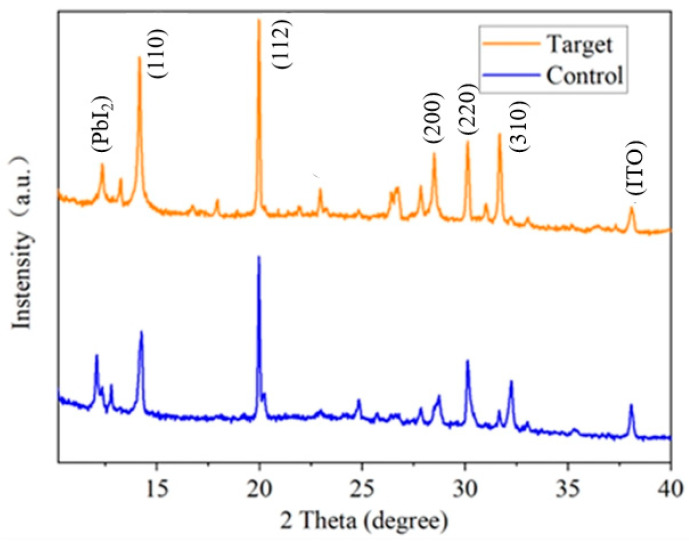
XRD patterns of chalcogenide films under different conditions.

To further verify the passivation effect of the KSCN + KCl dual additives on the WBG perovskite film, X-ray diffraction (XRD) spectroscopy measurements were performed in this study. In this work, the modified sample treated with the dual additives of KSCN (0.5 mol%) and KCl (1 mol%) was selected for comparison with the control. The XRD results of the WBG perovskite films before and after additive treatment are presented in Figure 6 below. The diffraction peaks of the perovskite films are observed at positions of 14.3°, 20.1°, 24.6°, 28.6°, and 32.1°, which can be assigned to the (110), (112), (202), (220), and (310) crystal planes of the perovskite phase, respectively. Several additional peaks, notably the one observed at approximately 38°, are attributable to the ITO substrate, as detected due to the penetration of X-rays through the perovskite film. As observed in the figure, the perovskite films (both before and after dual-additive treatment) exhibit the characteristic peaks of perovskite, with no significant shift in these peaks. This indicates that the dual additives do not induce lattice phase transition in the perovskite. The XRD peak intensity at 12.8° corresponding to unreacted PbI_2_ remained almost unchanged, suggesting that the passivation mechanism involves surface complexation, chemical precipitation, or ion exchange rather than disruption of the Pb crystal structure. The intensity of the (110) diffraction peak was significantly enhanced in the treated films, indicating higher crystallinity and more uniform grain alignment, consistent with SEM results. The decreased intensity ratio of the PbI_2_ peak to the perovskite (110) peak also suggests that the dual additives promote grain growth and perovskite formation.

### 3.2. Effect of KSCN and KCl Dual Additives on Defect Passivation in WBG Perovskite Films

**Figure 7:**
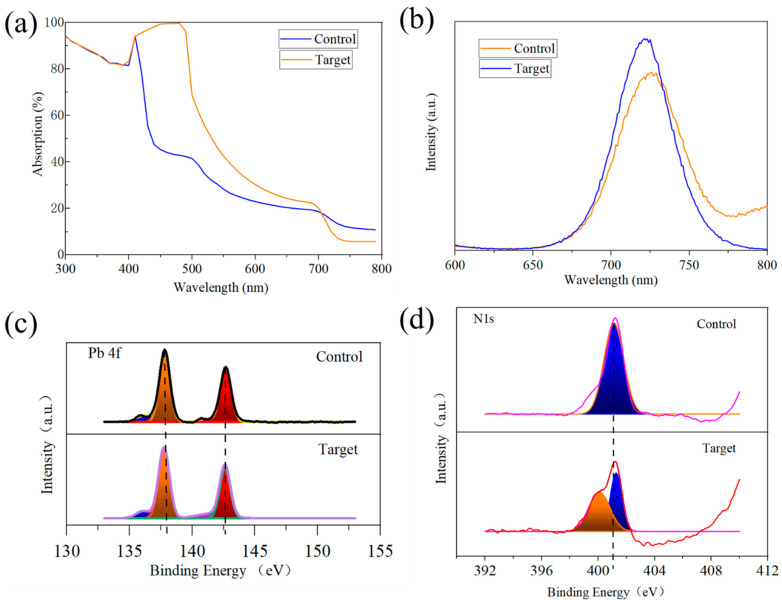
(**a**) UV-Vis absorption spectra of chalcogenide films before and after treatment with dual additives; (**b**) PL plots of chalcogenide films before and after additive treatment; (**c**) XPS spectra of Pb 4f before and after additive treatment and (**d**) XPS fine spectrum of N 1 s.

Optical performance measurements were conducted to characterize the quality of WBG perovskite films before and after dual-additive treatment, with a specific focus on the passivation effect of the dual additives on defects in the films. Ultraviolet-visible (UV-Vis) absorption spectra are presented in Figure 7a. The absorption capability of the treated films in the UV range (300~400 nm) was similar to the control, but a noticeable enhancement was observed within the visible spectrum (400~700 nm). This improvement is attributed to increased crystallinity and reduced non-radiative recombination due to defect passivation, leading to lower energy losses. Additionally, a slight blue shift in the absorption onset was observed for the treated films, indicating a slight increase in the optical bandgap of the WBG perovskite. This may be attributed to the strong coordination between SCN^−^ and Pb^2+^, causing local lattice distortion and strain, potentially shifting the valence band maximum upward or the conduction band minimum downward [33].

To demonstrate the changes in film quality, photoluminescence (PL) testing was conducted at room temperature. Using the same excitation conditions, compare the luminescence intensity and peak position of the samples. As shown in Figure 7b, the PL intensity of the WBG perovskite film increased by a factor of 1.2 after the introduction of KSCN + KCl dual additives. The enhanced PL signal signifies a higher radiative recombination rate, dominant over non-radiative pathways, due to effective defect passivation which reduces non-radiative recombination centers. A slight blue shift of the PL peak was also observed, consistent with the increased optical bandgap inferred from UV-Vis measurements.

To further characterize the passivation effect of dual additives on defects in perovskite films, X-ray Photoelectron Spectroscopy (XPS) measurements were performed in this study, and the experimental results are, leading to the contraction of Pb 4f orbitals and an increase in binding energy. A peak shift is observed in the XPS fine spectrum of N 1s (Figure 7d). In addition, the addition of KCl creates a competitive relationship with SCN^−^; Cl^−^ combines with Pb^2+^ to form PbCl_2_, further consuming uncoordinated Pb^2+^. This exacerbates the reduction in the electron cloud density around N atoms, thereby increasing the N 1s binding energy. Concurrently, due to the competitive effect of KCl, some SCN^−^ do not participate in coordination. These uncoordinated SCN^−^ may transfer electrons to N atoms in organic cations through the conjugation effect, which increases the electron cloud density of the N atoms, weakens the binding of the N atomic nucleus to inner-shell electrons, and results in the formation of a new peak with low binding energy.

**Figure 8:**
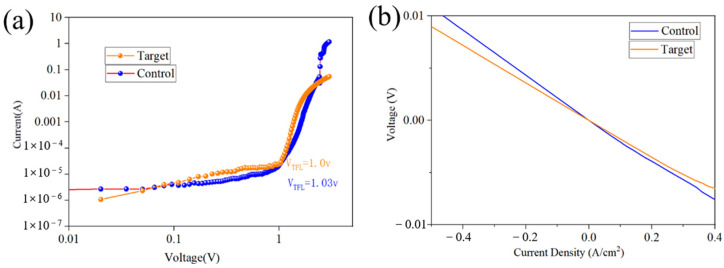
(**a**) Dark-state I-V plot; (**b**) Comparison of U-I curves before and after double additive treatment.

The carrier transport properties were characterized using the space-charge-limited current (SCLC) method (Figure 8a). Analysis of the dark current-voltage (I-V) curves revealed that the trap-filled limit voltage (*V_TFL_*) decreased from 1.06 V for the control device to 1.00 V for the device treated with dual additives. According to the defect density calculation formula (1), a decrease in *V_TFL_* signifies a reduction in the defect density within the perovskite film, further confirming the passivation effect.

Defect density is denoted by Formula (1) as:

(1) Nt=2ε0εrVTFLqL2

where ℰ_0_ represents the vacuum permittivity, ℰ*_r_* denotes the relative permittivity of the material, *V_TFL_* is the trap-filled limit voltage identified by the intersection point of the n = 1 and n > 3 regions in the current-voltage characteristic, *q* stands for the elementary charge (1.60 × 10^−19^ C), and *L* corresponds to the thickness of the perovskite film [34].

The electrical conductivity of the films was also measured (Figure 8b). The slope of the current-voltage (I-V) curve indicates that the film treated with dual additives exhibits superior conductivity. This suggests an increase in carrier concentration, confirms the reduction in non-radiative recombination, and indicates enhanced intrinsic recombination, further demonstrating effective defect passivation.

### 3.3. Effect of KSCN and KCl Dual Additives on the Performance of WBG Perovskite Solar Cells

**Figure 9:**
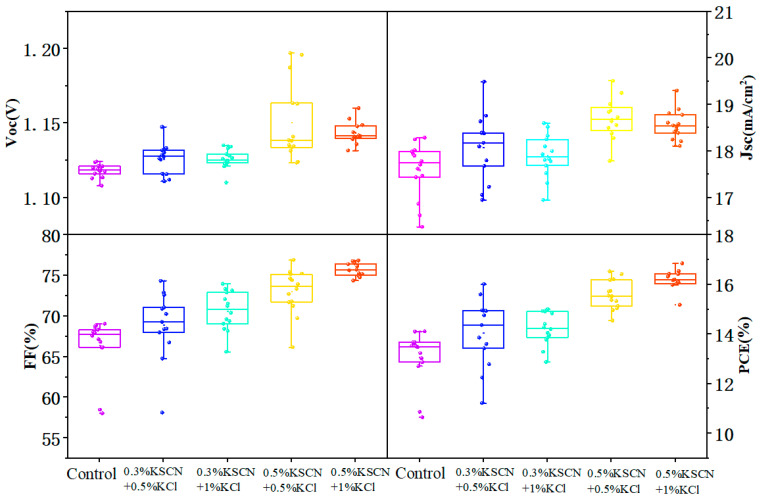
Statistical distribution of the effect of different concentrations of additive treatments on the performance of WBG chalcogenide solar cells.

The characterizations confirmed that the dual additives improve both crystallinity and reduce defect density. To validate this, single-junction inverted WBG PSCs were fabricated. Figure 9 compares the statistical distribution of photovoltaic parameters for control devices and those treated with different KSCN + KCl concentrations. For each fabrication condition (including the control and each dual-additive concentration), we prepared and characterized a total of 32 independent samples. The photovoltaic parameters across these samples showed a narrow distribution, and the statistical results consistently demonstrated the same trends in film properties and device performance. The champion devices treated with dual additives exhibited improvements in open-circuit voltage (*V_OC_*), short-circuit current density (*J_SC_*), fill factor (*FF*), and power conversion efficiency (*PCE*), demonstrating the significant beneficial effect. As can be seen from Figure 8 above, the champion device treated with dual additives exhibits improvements in open-circuit voltage (*V_OC_*), short-circuit current density (*J_SC_*), and fill factor (*FF*), which also leads to an enhancement in power conversion efficiency (*PCE*). Therefore, the dual additives of KSCN and KCl play a significant role in improving the device performance.

**Figure 10:**
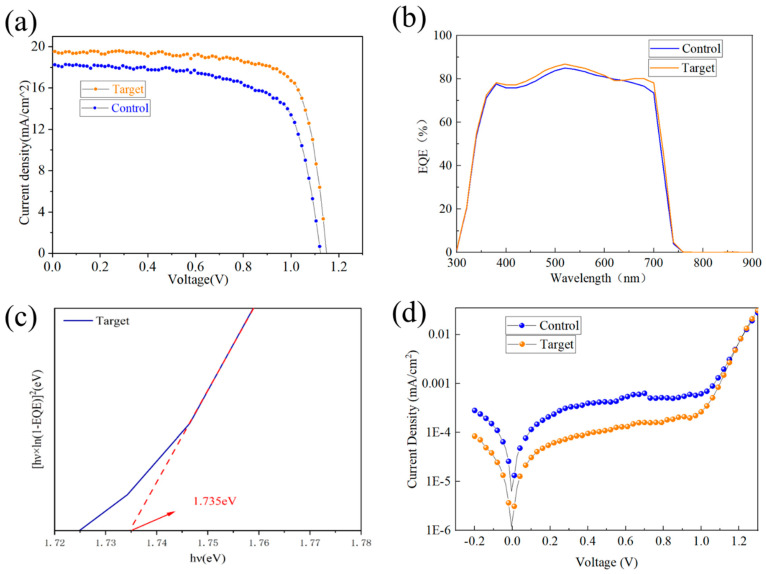
(**a**) *J-V* curves of Champion batteries without additive treatment and KSCN + KCl double additive treatment; (**b**) Plot of cell external quantum efficiency before and after additive treatment; (**c**) Calculated bandgap diagram of external quantum efficiency; (**d**) Dark *J-V* curves before and after additive treatment.

The maximum efficiency *J-V* curves of the control group and batteries treated with different concentrations of additives are shown in Figure 10a below. Among them, the control group champion cell exhibited a *V_OC_* of 1.117 V, a *J_SC_* of 18.29 mA/cm^2^, a *FF* of 69.05%, and a *PCE* of 14.11%. The champion cell treated with KSCN + KCl dual additives exhibited enhanced parameters. After treatment with dual additives KSCN + KCl, the champion cell exhibited a *V_OC_* of 1.148 V, a *J_SC_* of 19.51 mA/cm^2^, a *FF* of 75.17%, and a *PCE* of 16.85%. The performance enhancement indicates lower defect density in the battery, along with improved carrier transport capability and charge extraction efficiency, demonstrating the optimization potential of the KSCN + KCl dual-additive system for WBG perovskite films and cell performance.

The external quantum efficiency (EQE) spectra (Figure 10b) showed significantly higher values for the treated cell across the 400 nm~750 nm visible range compared to the control, indicating better utilization of incident light, higher carrier concentration, and improved charge transport, attributable to reduced defects and enhanced crystallinity. The bandgap estimated from the EQE spectrum was approximately 1.735 eV (Figure 10c), slightly increased compared to the literature value of 1.73 eV [35], consistent with PL and UV-Vis results.

Even though the passivation effect of the dual additives on perovskite defects can be demonstrated through battery performance, *J-V* curves, and EQE curves, we further analyzed changes in the internal defect density of the dual-additive-treated cells via dark-state *J-V* curve analysis under non-illuminated conditions. The test results are shown in Figure 10d. Compared to the control group, the battery treated with the KSCN + KCl dual additives exhibited a lower dark current density. This more directly indicates a reduction in the internal carrier recombination rate of the battery, reflecting a decrease in carrier recombination centers and an effective reduction in defect density.

The detailed parameters of the champion devices are summarized in Table 1. Compared to the control champion device, the champion device with KSCN (0.5 mol%) + KCl (1 mol%) dual additives showed improvements of 2.8% in *V_OC_*, 6.7% in *J_SC_*, 8.9% in *FF*, and ultimately 19.4% in *PCE*.

**Figure 11:**
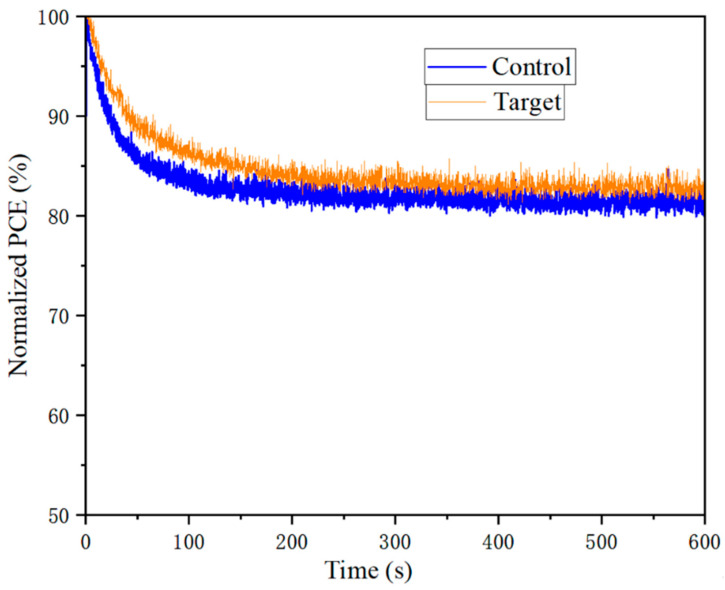
Effect on cell stability before and after dual additive treatment.

To investigate whether the introduction of dual additives can also enhance battery stability, maximum power point (MPP) tracking stability experiments were conducted. Experimental conditions were set at 23 °C and 30~40% RH. The time-dependent PCE evolution of the cells is shown in Figure 11. Although the stability measurement time is limited to 600 s due to instrument limitations, the figure also demonstrates that the champion cell treated with the dual additives exhibited a smaller PCE decline over the same time period, indicating superior stability compared to the untreated cell. Consequently, the introduction of the KSCN + KCl dual additives effectively suppresses photo-decoupling and enhances the cell’s light stability.

## 4. Conclusions

This study demonstrates that the incorporation of potassium thiocyanate (KSCN) and potassium chloride (KCl) as dual additives effectively enhances the photovoltaic performance and operational stability of wide-bandgap perovskite solar cells. Through systematic testing of various concentration combinations (KSCN (0.3 mol%) + KCl (0.5 mol%), KSCN (0.3 mol%) + KCl (1 mol%), KSCN (0.5 mol%) + KCl (0.5 mol%), and KSCN (0.5 mol%) + KCl (1 mol%)), the dual additives were found to significantly improve WBG perovskite film quality by promoting crystallization—evidenced by a 27.0% increase in average grain size, more uniform morphology, and reduced surface roughness—which collectively suppressed defect formation and non-radiative recombination. XRD results confirmed enhanced crystallinity without introducing phase impurities, while optical characterization showed strengthened visible light absorption despite a slight bandgap increase. Comprehensive characterization using PL, XPS, and SCLC measurements consistently revealed effective defect passivation, reduced defect density, a shift toward radiative recombination, and improved electrical conductivity. These synergistic improvements translated to exceptional device performance, with the champion device exhibiting a 19.4% increase in power conversion efficiency, along with a 2.8% increase in *Voc*, a 6.7% increase in *Jsc*, and an 8.9% increase in *FF* compared to the control, accompanied by significantly enhanced operational stability under continuous illumination. The results clearly highlight the considerable potential of the KSCN + KCl dual-additive strategy for developing high-performance and stable wide-bandgap perovskite photovoltaics.

We also note limitations such as the need for longer-term stability testing and deeper mechanistic studies on ion interactions.

Future work will explore stability under harsh conditions, investigate ion behavior with advanced characterization, and apply this approach to other perovskite types and tandem structures.

## Figures and Tables

**Table 1:** Parameters of KSCN + KCl Champion Cells with Different Concentrations.

Champion Devices	*V_OC_* (V)	*J_SC_* (mA/cm^2^)	*FF* (%)	*PCE* (%)	Active Area/cm^2^
Control	1.117	18.29	69.05	14.11	0.15
0.3 mol% + 0.5 mol% KCLKCl	1.131	19.49	72.63	16.02	0.15
0.3 mol% + 1 mol%	1.133	18.52	71.54	15.01	0.15
0.5 mol% + 0.5 mol%	1.187	18.46	75.41	16.53	0.15
0.5 mol% + 1 mol%	1.148	19.51	75.17	16.85	0.15

## Data Availability

The original contributions presented in this study are included in the article. Further inquiries can be directed to the corresponding authors.
